# The scrub typhus in mainland China: spatiotemporal expansion and risk prediction underpinned by complex factors

**DOI:** 10.1080/22221751.2019.1631719

**Published:** 2019-06-24

**Authors:** Hongwu Yao, Yixing Wang, Xianmiao Mi, Ye Sun, Kun Liu, Xinlou Li, Xiang Ren, Mengjie Geng, Yang Yang, Liping Wang, Wei Liu, Liqun Fang

**Affiliations:** a Department of Infection Management and Disease Control, Chinese PLA General Hospital, Beijing, People’s Republic of China; b The State Key Laboratory of Pathogen and Biosecurity, Beijing Institute of Microbiology and Epidemiology, Beijing, People’s Republic of China; c Center for Disease Control and Prevention of the North Military region, Jinan, People’s Republic of China; d Department of Epidemiology and Ministry of Education Key Lab of Hazard Assessment and Control in Special Operational Environment, School of Public Health, Fourth Military Medical University, Xi’an, People’s Republic of China; e PLA Strategic Support Force Characteristic Medical Center, Beijing, People’s Republic of China; f Division of Infectious Disease, Key Laboratory of Surveillance and Early-warning on Infectious Disease, Chinese Centre for Disease Control and Prevention, Beijing, People’s Republic of China; g Department of Biostatistics, College of Public Health and Health Professions, and Emerging Pathogens Institute, University of Florida, Gainesville, FL, USA; h Beijing Key Laboratory of Vector Borne and Natural Focus Infectious Diseases, Beijing, People’s Republic of China

**Keywords:** Scrub typhus, epidemiological feature, heterogeneity, spatiotemporal expansion, risk factors

## Abstract

In mainland China, a geographic northward expansion of scrub typhus has been seen, highlighting the need to understand the factors and identify the risk for disease prevention. Incidence data from 1980 to 2013 were used. A Cox proportional hazard model was used to identify drivers for spatial spread, and a boosted regression tree (BRT) model was constructed to predict potential risk areas. Since the 1980s, an invasive expansion from South Natural Foci towards North Natural Foci was clearly identified, with the epidemiological heterogeneity observed between two regions, mainly in spatial distribution, seasonality, and demographic characteristics. Survival analysis disclosed significant factors contributing to the spatial expansion as following: being intersected by freeway (HR = 1.31, 95% CI: 1.11–1.54), coverage percentage of broadleaf forest (HR = 1.10, 95% CI: 1.06–1.15), and monthly average temperature (HR = 1.27, 95% CI: 1.25–1.30). The BRT models showed that precipitation, sunshine hour, temperature, crop field, and relative humidity contributed substantially to the spatial distribution of scrub typhus. A county-scale risk map was created to predict the regions with high probability of the disease. The current study enabled a comprehensive overview of epidemiological characteristics of scrub typhus in mainland China.

## Introduction

Scrub typhus is a bacterial zoonosis caused by *Orientia tsutsugamushi*, which is mostly carried by rodents and chigger mites, and occasionally transmitted to humans by bites of infected larval mites [1,2]. *O. tsutsugamushi* is geographically endemic across broad areas of south and southeastern Asia, the Pacific islands, and northern Australia, with more than half (55%) of the world’s population being at risk for the disease [5]. In recent years, an obviously increased incidence of the disease has concurrently been documented from multiple Asia countries such as China, India, South Korea, Thailand and Laos, after a sporadic epidemic for several decades [3–8]. Under situation that no effective human vaccines or rapid laboratory diagnosis methods were available, scrub typhus continued to be a severe public health threat [3,9].

In China, although been identified as early as 1948, scrub typhus was limited in the tropics and subtropics areas of southern China for a long time period [10]. Only within last 3 decades the disease has emerged in northern China, e.g. Shandong, Jiangsu, and Anhui provinces [4,11]. Thereafter new natural foci were continuously reported and confirmed, resulting in a dramatically increased geographical distribution and an approximate 10-fold increase of the annual case number from 2006 to 2013 [12]. Temporal and demographic heterogeneity of the disease was preliminarily observed between northern and southern regions [13,14]. This has highlighted the need to understand what heterogeneous characteristics of the disease existed at the national scale, and which risk factors drove the recent spatial expansion of the epidemics.

The current study was designed to explore the geographic expansion and epidemiological characteristics of scrub typhus in different natural foci across a long time period in mainland China, based on which the environmental, socioeconomic, and meteorological factors favouring the occurrence and expansion of the disease were identified. We also sought to model scrub typhus transmission pattern and assess its risk distribution, with a goal of devising better targeted surveillance and control efforts in the future.

## Materials and methods

### Data collection and management

Disease incidence data of three sources were used for the current analysis. Firstly, all reported cases of scrub typhus from January 2006 to December 2013 were extracted from the China Information System for Disease Control and Prevention (CISDCP). The case definition was made according to the uniform diagnostic standard institutionalized by Chinese Ministry of Health (MOH) (http://www.chinacdc.cn/tzgg/200901/t20090105_40316.htm) (see supplementary material for detailed diagnostic criteria). For individual case, the data used for analysis included age, sex, occupation, and residence address at county level. The data before 2006 were acquired by two ways. The available historical data were collected from China CDC, which comprised the monthly number of reported cases from 1980 to 1989 at province level and the time of the first case reporting in each province from 1950 to 1980. The data before the CISDCP system running were collected by performing a complete literature search. All references involving the emergence of human scrub typhus cases were checked to document the exact year and location of the first case at province level [4,12,15–24].

All the data of cases used in this study were anonymized and the identity of any individual case cannot be uncovered. It was determined by the National Health Commission of China that the collection of data on scrub typhus cases was part of continuing public health surveillance of an infectious disease and was exempt from institutional review board assessment.

Data concerning environmental, socioeconomic, and meteorological factors were collected and used to explore potential determinants of the spatiotemporal distribution and spatial expansion of scrub typhus in mainland China. The area percentages occupied by croplands, forests and grassland in each county were extracted from the land cover data of China in 2005, available on the National Earth System Science Data Sharing Infrastructure (http://www.geodata.cn). Population data on the Sixth National Census in 2010 were obtained from the National Bureau of Statistics of China. Transportation routes were obtained from the digital maps of China at the scale of 1:100,000 as previously described [25]. Monthly meteorological data during the study period, including average temperature, relative humidity, sunshine hours, and precipitation were obtained from China Meteorological Data Sharing Service System (http://cdc.nmic.cn/home.do). Finally, a set of 15 variables were extracted for each county by using spatial analytic approaches in ArcGIS 9.3 software (ESRI Inc., Redlands, CA, USA).

### The analysis of spatiotemporal expansion and epidemiological features of scrub typhus

A thematic map was created to display the spatiotemporal expansion of scrub typhus in China. To explore the seasonal dynamic and long-term pattern of the disease, bar charts of monthly disease incidence combined with the annual incidence curves were respectively created for South Natural Foci (SNF: the Summer scrub typhus in southern provinces of Yangtze River) and North Natural Foci (NNF: the Autumn-winter scrub typhus in northern provinces of Yangtze River). Heat maps of monthly incidence for the two periods (1980−1989 and 2006−2013) were created to present the province-specific temporal dynamics, and further related to the latitude of each province from north to south of mainland China [26].

### The analysis of risk factors associated with the spatial expansion of scrub typhus

Totally 15 environmental, socioeconomic and meteorological factors (Supplementary Table 1) were evaluated for their contribution to the spatial expansion of scrub typhus. A Cox proportional hazard model was applied with the county-level invasion time in years (interval from the year when the first case was confirmed to the year of 2006) used as outcome. Unaffected counties until the year of 2013 were considered as right-censored. Hazard ratios (HR) for the continuous variables were calculated.

Univariate analysis was performed to examine the effect of each individual variable. Multivariate analysis started by including the variables with *P*-values of <.2 in the univariate analyses, and the final model was reached by excluding variables with *P*-values of > .05. The collinearity of variables was devoid by removing the one with less significance from the model. Statistical analyses were performed using the Stata 9.1 package (StataCorp LP, College Station, TX) [27].

### Risk assessment of the spatial distribution of scrub typhus

The boosted regression tree (BRT) models were separately constructed for NNF and SNF. For both models, the land cover and meteorological variables were used as predictors for the occurrence of scrub typhus cases (Supplementary Table 1). For the specific numbering of the variable, we set a tree complexity of 5, and a learning rate of 0.005 and a bag fraction of 75%. We employed a “case-control” design, with all counties reporting scrub typhus cases considered as “cases,” while those without case reporting as “controls.” The case-control ratios were sampled as 1:4 in northern regions and 1:1 in southern regions based on their epidemic extending. Seventy-five per cent points from 2006 to 2012 were randomly selected and set as training datasets while the remaining 25% points were used as test datasets. Points in 2013 were used for prediction and predictive power estimation. The model-fitted risks were plotted on each predictor. Furthermore, receiver-operating characteristic (ROC) curves and areas under the curve (AUC) were produced to determine the predictive power of the models. Based on the predicted probabilities, a risk map for scrub typhus was created. We further estimated the number of population distributed in risk regions based on the risk map. The estimated population value was calculated based on the risk map derived from the mean prediction across all 50 BRT ensembles. The number of people living in areas that are suitable for scrub typhus transmission was also estimated. All statistical analyses were performed with the R 3.1.1 software, and dismo and gbm packages were used.

## Results

### Spatiotemporal expansion and epidemiological features

#### Spatiotemporal heterogeneity between NNF and SNF

**Figure 1.**
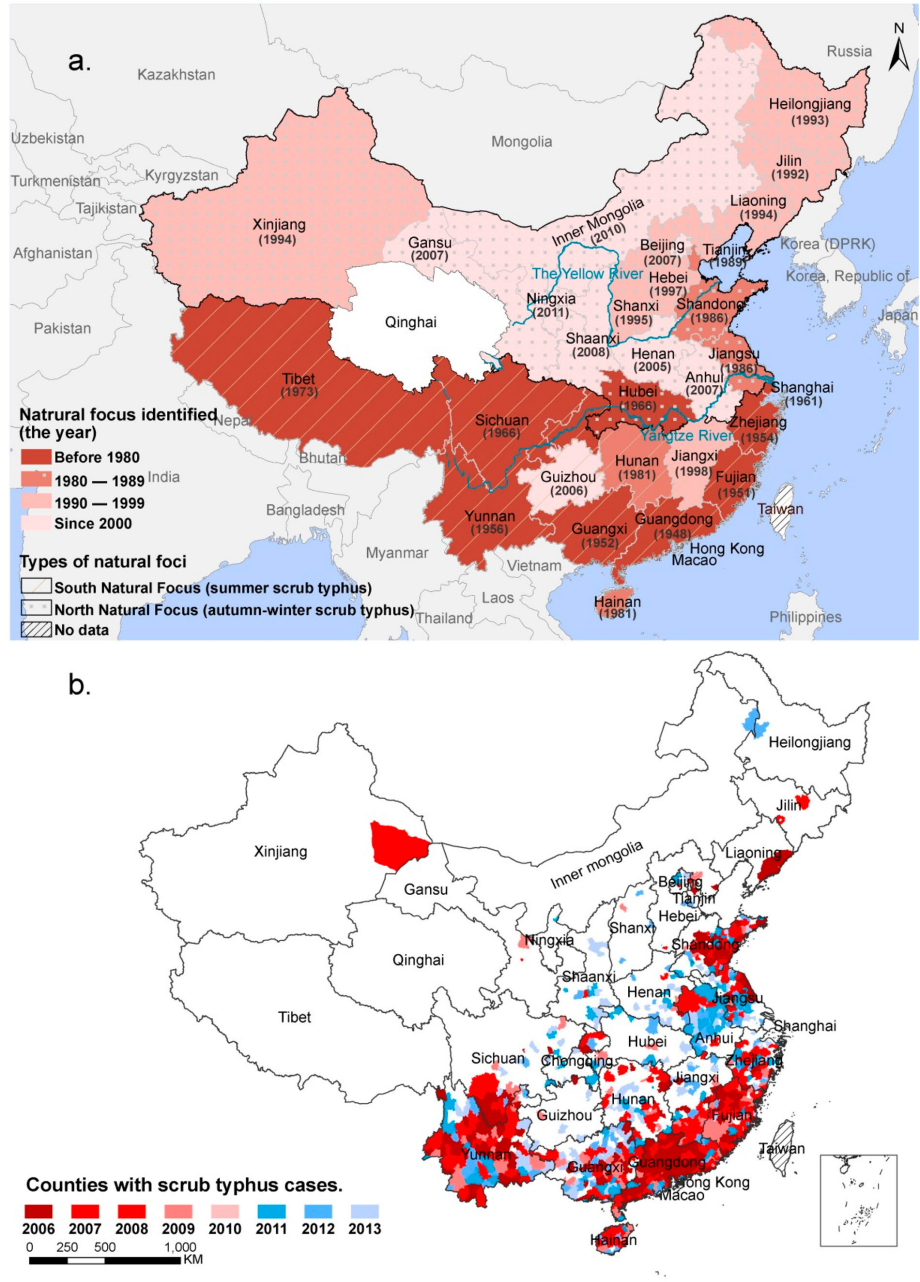
The spatial dynamic of scrub typhus in China. (a) The historical spatial dynamic of scrub typhus in China with the year of natural foci first identified in each province. (b) The spatial dynamic of scrub typhus in China from 2006 to 2013. Note: Hainan Province was established in 1988; Chongqing Municipality was established in 1997.

Scrub typhus was largely limited in South China before the 1980s. In 1986, the first record of the disease outbreak in Mengyin County of Shandong Province and Dongtai County and Nanjing City of Jiangsu Province seemed to open a rapid northern invasion way that crossed Yangzi River, initiating an expedited expansion towards northern China starting from the 1980s (Figure 1(a)). By 1990s, the reporting provinces had extended over latitude 44°N with the majority of provinces having reported the disease in mainland China. The recent data also displayed that natural foci of scrub typhus continued to expand northwards, with the disease extending to the most northern part in Aihui County of Heilongjiang Province (Figure 1(b)). Collectively a three-fold increase of the case-reporting counties from 222 in 2006 to 691 in 2013 was determined.

A surge of disease incidence was likewise observed between 2006–2013 in comparison with 1980–1989 period. A total of 36,081 clinical-confirmed and 2522 laboratory-confirmed scrub typhus cases were reported from 28 provinces in China from 2006 to 2013. In SNF, the increases were from 1.85 to 4.41 in 1980–1989 and from 1.53 to 15.91 in 2006–2013, while in NNF, the increases were from 0 to 0.77 in 1980–1989 and from 0.54 to 3.01 in 2006–2013. These had remarkably broadened the incidence discrepancy between two regions.

**Figure 2.**
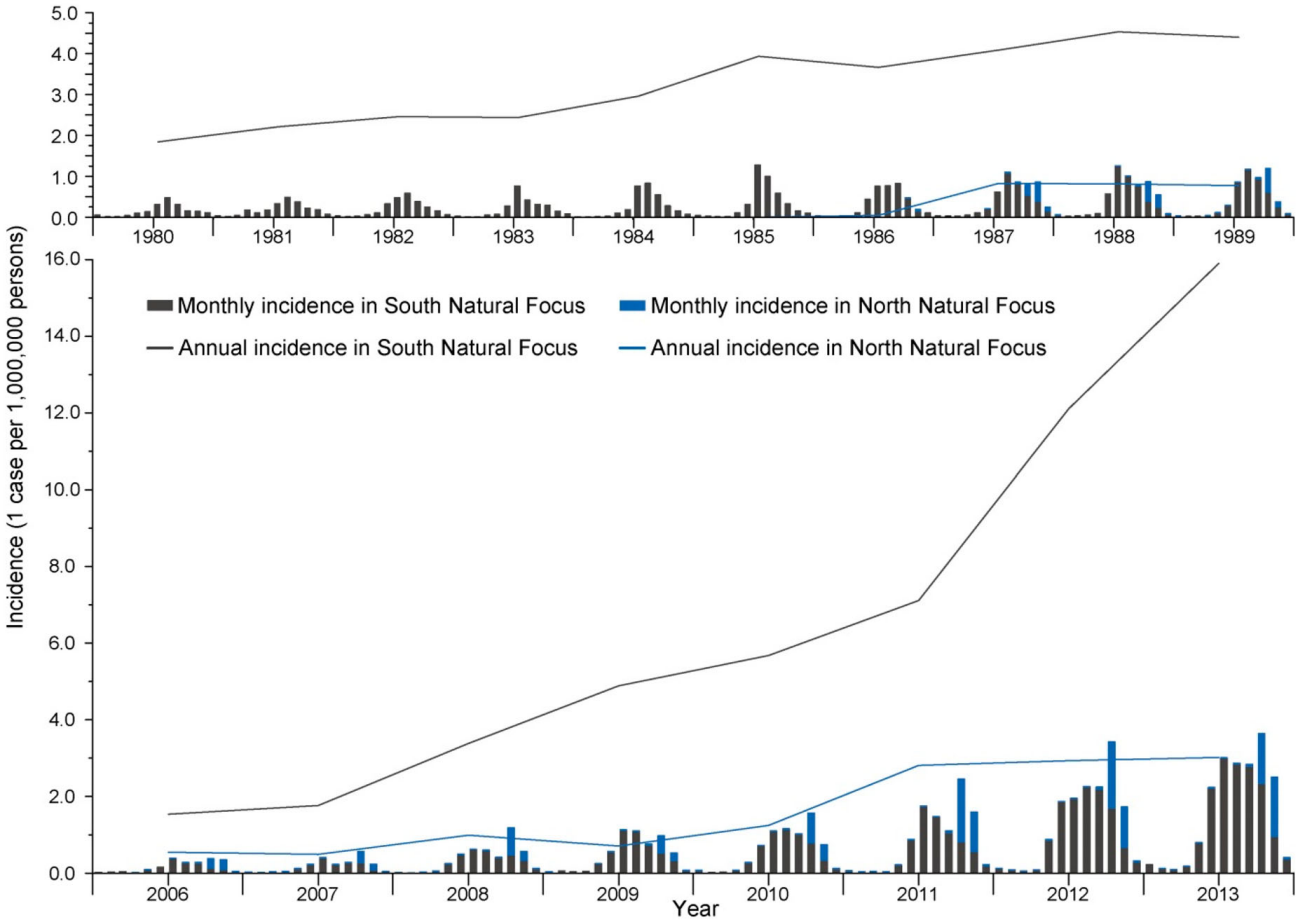
Temporal distribution of the scrub typhus incidence in mainland China The gray and blue histograms represented the monthly incidence of South and North Natural Foci, while the gray and blue curves represented the annual incidence of South and North Natural Foci separately.

**Figure 3.**
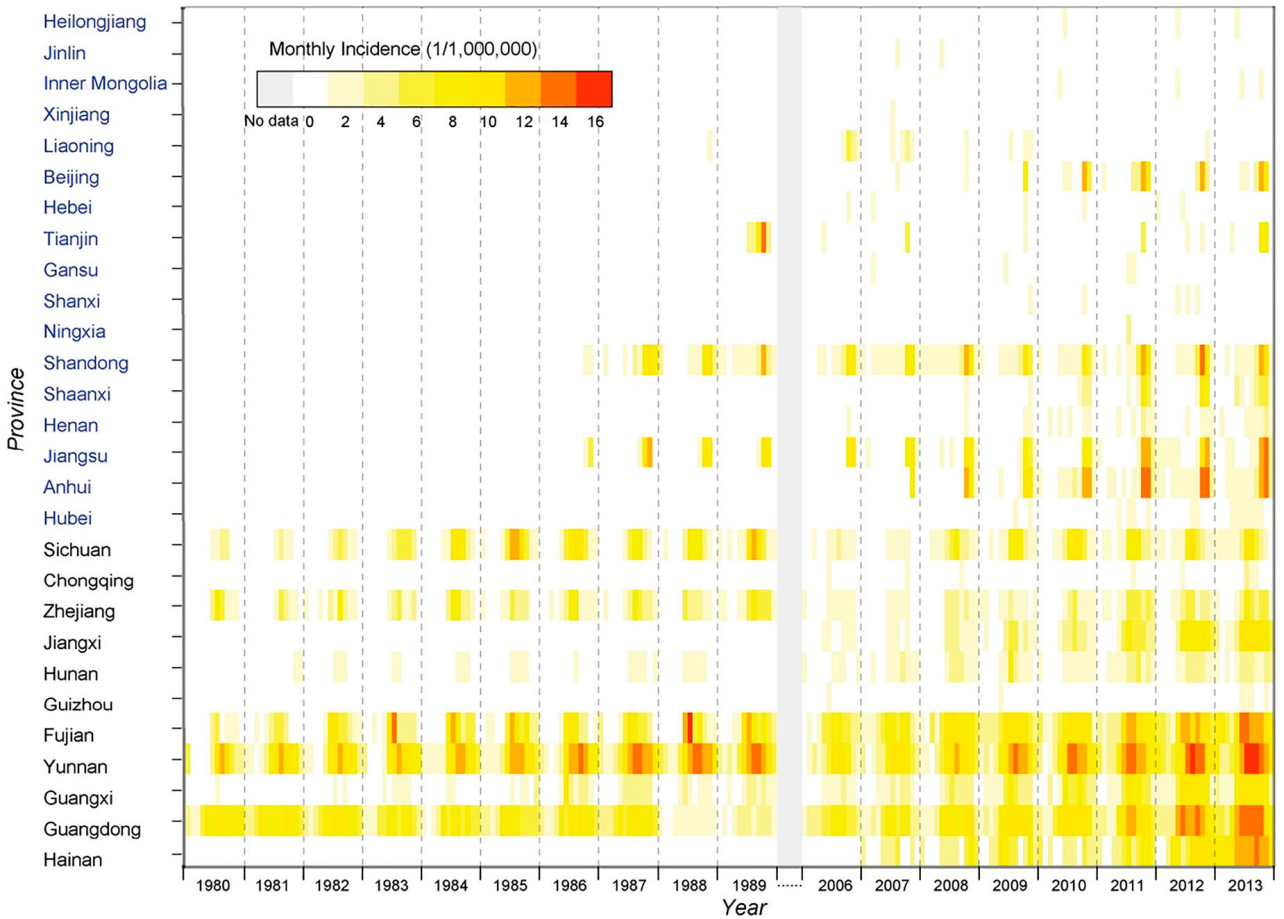
Heat map of monthly incidence of each involved province. Monthly incidences of all involved provinces were shown in heat map ordered from north to south.

**Figure 4.**
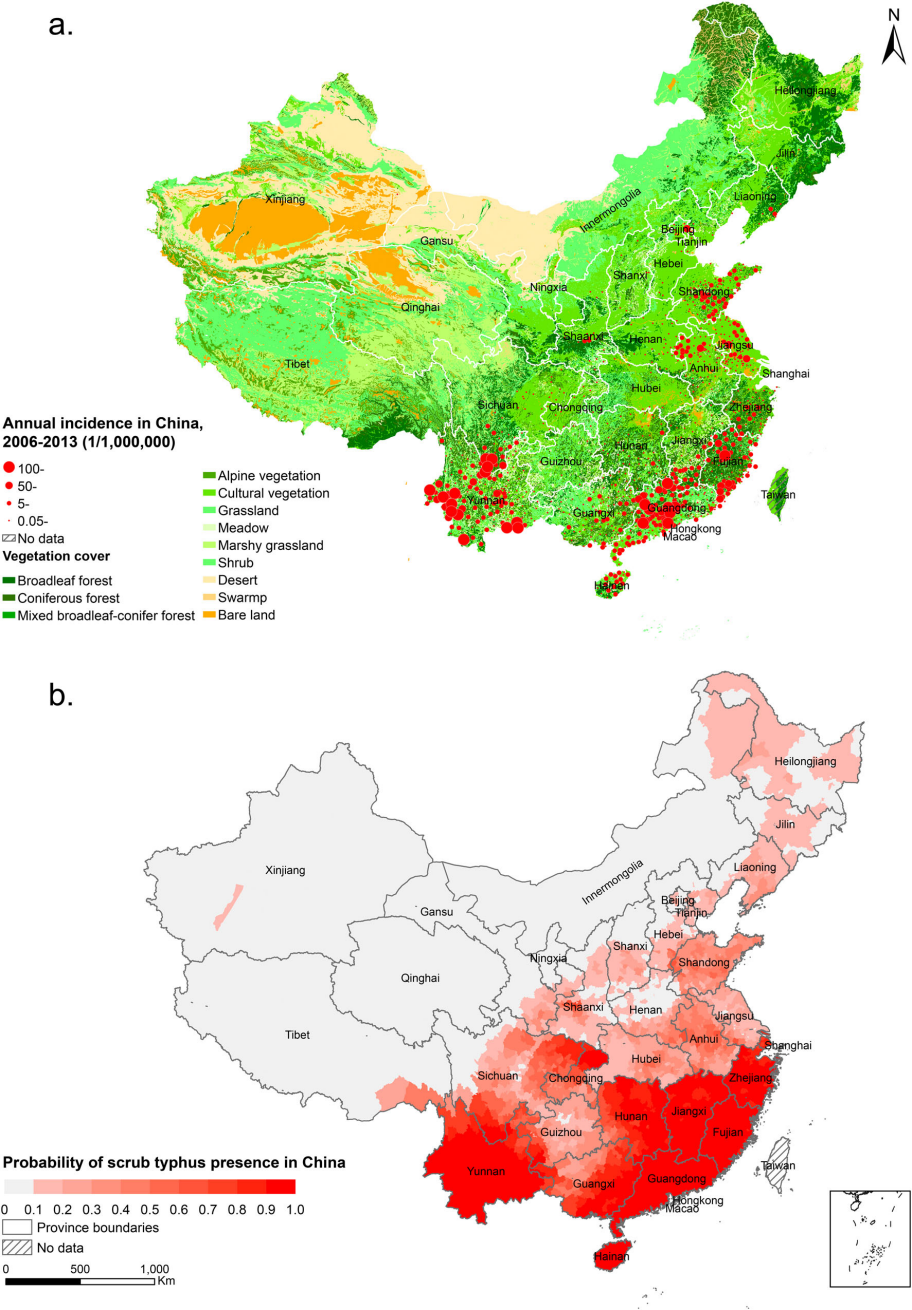
The spatial distribution of scrub typhus in China. (a) The distribution of annual incidence in mainland China from 2006 to 2013. (b) The predicted risk distribution of scrub typhus at the county level in mainland China.

Generally, the seasonal heterogeneity of the disease was obvious between the two foci (Figure 2). In SNF, a temporal peaking incidence was noticed in summer (July–September), which was observed with one to three months lagging in NNF. The disease epidemic peak shifted obviously from summer to autumn with the latitude level increased for each province, which corresponded with a late epidemic of the disease in northern endemic provinces (Figure 3). A thematic map displayed a wide range of the average annual incidence at the county level, with the most severe endemic areas observed in provinces along the coast of Southern and Eastern Chinese Sea, followed by Yunnan Province in southwestern China and northern Anhui Province in eastern China (Figure 4(a)).

#### Demographic heterogeneity between NNF and SNF

The median age of the patients was 50 years old (interquartile range: 33–61), with 68.54% of cases aged 40 and above, indicating increased odds of acquiring infection as getting old. The highest annual average incidence was documented in the patients of older than 60, which was observed with a similar trend across studied years (Supplementary Fig. 1). More female cases were reported, with a female-to-male ratio of 1.18:1 in NNF and 1.06:1 in SNF. The average annual incidence of female was also higher than male in both NNF (1.72/1.39) and SNF (6.97/6.16). Among the age group of 0–9 years old in both foci and the 10–19 years group in SNF, male had higher incidence rate than the female, which trend was reversed at group aged 20–60 years old in NNF and group older than 40 years in SNF (Supplementary Fig. 2). Moreover, the comparison between two foci demonstrated discrepancies for the age and occupation, as well as rural distribution pattern. The children under 10 years old had a significantly higher incidence in SNF than the same age group in NNF (6.98 vs. 0.45, *χ*^2^ = 4169.8, *P *< .001), and also displayed a rapid increase across the observed years (Supplementary Fig. 1 and Supplementary Fig. 2). Totally, the occupation of farmer had taken over half of the total patients, with the proportion increased from 58.5% in 2006 to 70.7% in 2013 (Supplementary Fig. 2). Interestingly, farmer occupation was significantly higher in NNF than that in SNF (84.7% vs. 63.1%, *χ*^2^ = 1533.4, *P *< .001). As has been reflected by the age-specific incidence, the scattered and preschool children had taken a second highest proportion, significantly higher in SNF than in NNF (10.6% vs. 3.2%, *χ*^2^ = 483.1, *P *< .001). In NNF, higher case incidence was reported in urban areas than in rural areas (1.61 and 1.54), which was significantly (*χ*^2^ = 326.0, *P *< .001) different from that of SNF (5.62 in urban and 7.43 in rural areas) (Supplementary Fig. 3).

### Risk factors associated with the spatial expansion of scrub typhus

The risk factors associated with spatial expansion of scrub typhus were determined by applying survival analysis, and the interval from the year when the first case was confirmed to the year of 2006 used as outcome for each county. Altogether 13 factors were significantly associated with the invasion of scrub typhus (Table 1). Only three factors remained in the multivariate analysis, including being intersected by freeway (adjusted HR = 1.31, 95% CI: 1.11–1.54, *P *= .001), coverage percentage of broadleaf forest (adjusted HR = 1.10, 95% CI: 1.06–1.15, *P *< .001), and monthly average temperature (adjusted HR = 1.27, 95% CI: 1.25–1.30, *P *< .001), indicating their significant contribution to the expansion of scrub typhus.

**Table 1.** Association between invasion time of scrub typhus and influencing factors at the county level in survival analysis, from 2006 to 2013. The invasion time denotes the interval from the year when the first human case of scrub typhus was confirmed to the year of 2006.

Variables	Univariate Cox analysis		Multivariate Cox analysis	
	Crude HR (95% CI)	*P*-value	Adjusted HR (95% CI)	*P*-value
Distance to the nearest epidemic county	0.74 (0.70, 0.78)	<.001	NS (excluded)	
Intersected by national highway	1.12 (0.95, 1.31)	.175		
Intersected by freeway	1.82 (1.54, 2.13)	<.001	1.31(1.11, 1.54)	.001
The proportion of flow population	1.03 (1.01, 1.04)	.001	NS (excluded)	
Coverage percentage of coniferous forest	1.23 (1.18, 1.29)	<.001	NS (excluded)	
Coverage percentage of mixed broadleaf-conifer forest	0.96 (0.87, 1.05)	.368	NS (excluded)	
Coverage percentage of broadleaf forest	1.29 (1.25, 1.34)	<.001	1.10 (1.06, 1.15)	<.001
Coverage percentage of shrub	1.08 (1.03, 1.14)	.004	NS (excluded)	
Coverage percentage of grassland	0.74 (0.69, 0.80)	<.001	NS (excluded)	
Percentage coverage of dry field	0.90 (0.87, 0.93)	<.001	NS (excluded)	
Percentage coverage of paddy field	1.18 (1.14, 1.22)	<.001	NS (excluded)	
Monthly cumulative precipitation	1.33 (1.30, 1.37)	<.001	NS (excluded)	
Monthly average temperature	1.29 (1.27, 1.32)	<.001	1.27 (1.25, 1.30)	<.001
Monthly average relative humidity	1.85 (1.76, 1.95)	<.001	NS (excluded)	
Monthly average sunshine hours	0.31 (0.26, 0.38)	<.001	NS (excluded)	

### Risk prediction on the spatial distribution of scrub typhus

Altogether 10 factors were further estimated for their roles in the spatial distribution of scrub typhus by using the BRT models, with the weight of all factors summed to 100%. Models were constructed for the two foci separately. In NNF, the significant contributing factors included precipitation, sunshine hour, temperature, percentage coverage of crop field and relative humidity (Table 2), while in SNF, only sunshine hour, temperature, and relative humidity have significantly contributed to the distribution of the disease. The meteorological factors were found to be the main impacting factors in NNF, with precipitation and sunshine hour overweighting the others (mean relative contribution 33.70 and 20.04, respectively). While for SNF, the infection risk was more likely to be associated with temperature (mean relative contribution 51.74).

**Table 2.** Summary of the relative contributions of predictive variables for the scrub typhus occurrence in the boosted regression tree model.

Variables^a^	Relative contributions (SD), %	
	NNF	SNF
Precipitation	33.70^a^ (2.24)	4.79 (1.13)
Sunshine hour	20.04^a^ (2.05)	15.14^a^ (1.21)
Temperature	14.87^a^ (2.31)	51.74^a^ (2.16)
Relative humidity	6.78^a^ (1.36)	6.32^a^ (0.93)
Percentage coverage of crop field	11.89^a^ (2.33)	3.79 (0.94)
Percentage coverage of broadleaf forest	3.93 (0.90)	3.29 (0.73)
Percentage coverage of coniferous forest	2.29 (0.65)	3.15 (0.61)
Percentage coverage of mixed broadleaf-conifer forest	1.55 (0.88)	2.65(0.76)
Percentage coverage of grassland	3.35 (0.78)	4.32 (0.86)
Percentage coverage of shrub	1.59 (0.44)	4.63 (0.79)

Note: SD: Standard Deviation. NNF: North Natural Foci. SNF: South Natural Foci.

^a^Variables with relative contribution >5 in the BRT models were considered as significant contributors to the occurrence of scrub typhus.

The model-fitted risks were plotted on each predictor, showing their non-linear relationships with scrub typhus incidence for both epidemic foci. The environmental and meteorological factors that favoured the disease distribution also differed between two foci (Supplementary Fig. 4). In NNF, precipitation above 400 mm, sunshine hour ranging 140–180 h, temperature ranging 9–14°C, percentage coverage of crop field ranging 47–80%, and relative humidity ranging 62–65% contributed to the risk of scrub typhus development. In SNF, temperature over 15°C, and sunshine hour around 150 h, as well as relative humidity below 63% were more favourable for the disease spread.

In the model for NNF, the AUC value was 0.978 (95% CI: 0.971–0.984) for the training dataset, 0.916 (95% CI: 0.900–0.932) for the test dataset, and 0.838 (95% CI: 0.812–0.864) for the prediction, which performed better than the model for SNF, where the AUC value was 0.911 (95% CI: 0.892–0.931) for the training one, 0.907 (95% CI: 0.888–0.925) for the test one and 0.841 (95% CI: 0.819–0.863) for the prediction. All these results suggested decent predictive accuracies of the models.

The risk map was plotted based on the model (Figure 4(b)). In SNF, a high risk of scrub typhus disperse was determined in Yunnan, Guangdong, Hainan, Fujian, Guangxi, Zhejiang and south of Sichuan, consistent with the observed endemic, all being old endemic regions with long histories of scrub typhus epidemic. Additionally, the map revealed neglected endemic regions such as Hunan, Jiangxi, north of Chongqing and northeast of Sichuan, where a high probability of scrub typhus incidence was demonstrated. Most emerging foci were predicted to lie in NNF, such as Hebei, Liaoning, Jilin, Heilongjiang, south of Gansu, and west of Xinjiang, where intense surveillance efforts are needed. Taken together, we estimated that 192.351 (95% CI: 187.105–195.745) million people lived in the potential infection areas of scrub typhus in mainland China, with majority (70.84%) of infection concentrated in five provinces (Guangdong, Jiangsu, Fujian, Guangxi and Yunnan).

## Discussion

In this study, by using long period disease data, we comprehensively assess the national distribution of scrub typhus for the first time. In addition to show a clear cut northward expansion of the disease that has been displayed in some provinces [14,28–30], we firstly revealed epidemiological characteristics and risk factors that were differentially established between NNF and SNF. We also constructed high-resolution nation-wide risk map, using an established modelling framework that combined an integrated database comprising meteorological conditions, environmental and socioeconomic factors, in determining the disease emergence.

A complete description of the geographic expansion of scrub typhus in mainland China was provided in the current study. With the expansion of primal foci in southern China and the emergency of new foci in northern China, the traditional geographic bordering of the disease no longer exists, thus the medical care workers should make fully referring to the scrub typhus in treating the patients with fever of unknown reason.

The seasonal discrepancy was found between NNF and SNF, which were respectively featured as Autumn-winter and Summer scrub typhus foci. On one hand, dominant vectors and animal hosts of the disease differed among the geographic locations, e.g. *Leptotrombidium scutellare* is the most important vector responsible for the transmission of the disease in the northern area of Yangtze River, while *Leptotrombidium deliense* and *Leptotrombidium gaohuense* predominant in the southern area of Yangtze River [31–34]. The main animal hosts are *Apode musagrarius* and *Cricetulus triton* in the north, while *Rattus losea*, *Rattus flavipectus* and *Rattus norvegicus* in the south, respectively [31–34]. On the other hand, discrepancy of human–environment interactions between two regions might also be responsible. For example, more people tend to be engaged in outdoor activities during summer vacation in the southern region [30], which increases the chance of people being exposed to scrub typhus in summer in southern provinces, while the patients in northern provinces are more prone to be exposed during autumn-winter. All these differences might contribute to the differential epidemiological characteristic.

We also identified differences in demographic characteristics, including age, gender and occupation. An age-dependent incidence was obvious, which is consistent with previous findings showing that the elderly were more likely to be infected than the young [35–37]. For one thing, boys are more likely to play outside, thus having more chance to be exposed to the infected vectors or animals than girls. The situation changed for adult population, as in rural areas, female had turned into the major working labour and taken most of the farming work in the field. All these factors were assumed to play roles by altering the outdoor activity, eventually impacting on the human contact frequency with the vectors. Unexpectedly, children under 10 years old were at increased risk of disease in recent years than previously recorded [12], especially in southern provinces. Scattered and preschool children ranked second on the occupation list in both foci, implicating children as a neglected group, for whom the education and preventive measures need to be intensified.

The spatial expansion and survival analysis demonstrated that counties being intersected by freeway, with high percentage coverage of coniferous forest and high average temperature were associated with the spatial expansion (Table 1). This is biological feasible, as the population density of vector or animal hosts in suitable habitat and its contact frequency with human being might be affected by these variables [38]. Anthropogenic factors in the movement of animal hosts, especially increasing trade of agricultural products and domestic animals along the highways cannot be ignored as well.

A previous work has pointed to a tight link between case number of vector-borne diseases and hot weather [39]. The same mechanism could offer a credible explanation of these associations, although the vector might differ among various vector species. In SNF, temperature had multiple effects on the disease, both via promoting the larvae and rodent density, and by increasing the exposure of victims with lessening clothes and frequenting outdoor activities. In Korea, an annual average temperate of 10°C was estimated to be a northern limit for the distribution of *L. scutellare*, which was also important chigger mite vector in many provinces in mainland China especially in northern provinces [35]. Under the same situation, the temperature limit, which has restricted the activity scope of the competent vectors, was no longer practical in setting the bordering of the disease, as the global warming might obviously promote the expansion of its habitat to the north, as had been exactly displayed from the current research. We propose that temperature as the most important environmental factors, along with aggravated greenhouse effect and global warming lately, had largely accounted for the spatial expansion towards the northern provinces in mainland China. As has been noted by previous research, the actual situation of global warming in China was mainly present in north of the 35°N in China [40], therefore contributing with more effects to the disease increase in NNF.

Additionally, the precipitation and sunshine hour overweighting other factors were found to play main roles in determining the disease incidence in NNF. While for the SNF, the risk was more likely to be associated with temperature (Table 2). It’s biological reasonable since temperature and rainfall might both directly alter the human behaviour and indirectly change the chigger abundance [41].

Precipitation was found to be positively associated with scrub typhus incidence in NNF. It’s postulated that rain might have promoted an increase in the chigger population, hence increasing the risk of transmission of scrub typhus when farmers harvested crops. Most importantly, humidity was considered as a determinant, for the mites’ density, as its survival relied on the water vapour for water source [35,42]. However, higher humidity was also unfavourable to the life cycle of the mites. As we have found in the current study, that areas with relative humidity higher than 63% would be at lower risk than others in SNF. Zheng et al.’ study also found that relative humidity was negatively associated with the occurrence of scrub typhus, which differed from the expectations that the risk is increased in wet environments hosting a higher abundance of mites [18]. Therefore, a detailed surveillance of the humidity of an environmental niche might help to offer a fine mapping of future incidence of the disease. Sunshine was reported to be one of the defining factors of larval activity hour [42], which might partially explain the lower disease incidence in northern than southern China. In north, sunshine hour below 170 turned to be positively, while over 170 negatively related to the disease in the BRT model, whereas the results differed in SNF. Consistently, Yang et al.’s study had proved sunshine hour to be a negative factor using Spearman’s correlation analyses in Shandong, north of Yangtze River in mainland China [43].

Since larvae population densities were highest in areas of increasing substrate vegetation [42], land type could be an important variable in making prediction. We disclosed different effect of land type from two foci. In NNF, the crop field contributed most significantly among all land types, while in SNF shrub and grass land played the most important part. A favourable habitats crop fields meant enhanced farming activities that were related to the high risk of exposure for farmers [44]. Apart from cultivated vegetation, natural vegetation, especially from grassland and shrub in south provinces, contributed with significance to the enhanced disease occurrence. Under the eco-friendly situation in urban areas, the extension of City Park would offer higher odds of acquiring scrub typhus for citizens [45], probably leading to the urbanization of scrub typhus which had already been alerted in Korea [46].

The risk map for scrub typhus in mainland China unfolded high risk areas in the south foci of Yangtze River including Yunnan, Guangdong, Guangxi, Hainan and Fujian; while in the north foci of Yangtze River, warning should be enhanced in Jiangsu, Anhui and Shandong. Imperative alert should be raised in regions, including Chongqing, Sichuan, Hubei, Shaanxi, Shanxi, Beijing, Tianjin, Liaoning, Jilin, Heilongjiang and Inner Mongolia. Actually natural foci of scrub typhus in Beijing had been confirmed for the first time very recently [37], further corroborating our appeal for future surveillance and control measures.

The study was subject to major limitation that only cases who sought medical care in hospital were reported to local CDC and used for analysis, therefore the underreport of clinical cases or subclinical infection who failed to see doctors were missed from the current analysis. On the other hand, important factors, including the mite prevalence data and pathogen genotype, both of which might be associated with the epidemiological heterogeneity, were not obtained for the current analysis due to the lack of field survey.

Despite of all these limitations, the current findings enable a comprehensive comparison of epidemiological characteristics of scrub typhus between North and South natural foci, supporting a complex mechanism that underlies disease incidence in China. The derived risk map provides a baseline for identifying areas where prevention and control efforts should be prioritized in the future.

## Supplementary Material

Supplemental Material
