# Supplementary material for: The scrub typhus in mainland China: spatiotemporal expansion and risk prediction underpinned by complex factors
Source: Emerg Microbes Infect. 2019 Jun 24;8(1):909–19. doi: 10.1080/22221751.2019.1631719 (PMC6598543; doi:10.1080/22221751.2019.1631719)
Supplement: Supplemental Material [file TEMI_A_1631719_SM6646.zip › Supplementary Material/temi-2019-0157-20190611180909/doc/Supplementary_material.docx]

**Supplementary material**

**The Scrub Typhus in mainland China: spatiotemporal expansion and risk prediction underpinned by complex factors**

**Diagnostic criteria of scrub typhus**

A probable case of scrub typhus is defined as meeting the following criteria: epidemiological exposure histories (travelling to endemic areas or contact with chiggers or rodents within three weeks prior to the onset), clinical manifestations (acute fever, skin rash, eschars or ulcers, and lymphadenopathy), and an agglutination titer ≥ 1:160 in the Weil-Felix test using the OXK strain of Proteus mirabilis. A laboratory-confirmed case is defined as a probable case with at least one of the laboratory criteria: a fourfold or greater rise in serum IgG antibody titers between acute and convalescent sera detected by using indirect immunofluorescence antibody assay (IFA), detection of *O. tsutsugamushi* by polymerase chain reaction (PCR) in clinical specimens, or isolation of *O. tsutsugamushi* from clinical specimens.

**Figure Legends**

**Supplementary Fig. 1 Age-gender and occupation distribution of the incidence (1/1,000,000) of scrub typhus in North and South Natural Foci, from 2006 to 2013.**

*: χ^2^ test, *P*<0.05.

**Supplementary Fig. 2 Temporal distribution of the scrub typhus incidence (1/1,000,000) of each age group in North and South Natural Foci, from 2006 to 2013**.

**Supplementary Fig. 3 Temporal trend of urban-rural distribution of the incidence of scrub typhus in North and South Natural Foci, from 2006 to 2013.**

**Supplementary Fig. 4 Relationships between risk factors and scrub typhus occurrence in the BRT models**. **a** For North Natural Foci, the scrub typhus risk functions of the BRT models is plotted as a function of precipitation, sunshine hour, temperature, percentage coverage of crop field, relative humidity, and others. **b** For South Natural Foci, the scrub typhus risk functions of the BRT models is plotted as a function of temperature, sunshine hour, relative humidity, precipitation, percentage coverage of shrub and others. The curves are average predicted lines for above variables by 50 repeats based on the bootstrapping procedure.
