# Supplementary material for: The scrub typhus in mainland China: spatiotemporal expansion and risk prediction underpinned by complex factors
Source: Emerg Microbes Infect. 2019 Jun 24;8(1):909–19. doi: 10.1080/22221751.2019.1631719 (PMC6598543; doi:10.1080/22221751.2019.1631719)
Supplement: Supplemental Material [file TEMI_A_1631719_SM6646.zip › Supplementary Material/temi-2019-0157-20190611180909/doc/Supplementary_Table_1.docx]

**Supplementary Table 1.** Description of potential influencing factors used in the analyses.

| **Variables** | **Description (Unit)** | **Type** |
| --- | --- | --- |
| The distance to the nearest epidemic county | The distance from a county to the nearest epidemic county (100km) | Continuous |
| Intersected by national highway | A county if it is intersected by national highways | Binary |
| Intersected by national freeway | A county if it is intersected by national freeways | Binary |
| The proportion of flow population | The proportion of flow population in the total population for each county (10%) | Continuous |
| Percentage coverage of coniferous forest | Percentage coverage of coniferous forest for each county (10%) | Continuous |
| Percentage coverage of mixed coniferous-broadleaf forest | Percentage coverage of mixed coniferous-broadleaf forest for each county (10%) | Continuous |
| Percentage coverage of broadleaf forest | Percentage coverage of broad leaf forest for each county (10%) | Continuous |
| Percentage coverage of shrub | Percentage coverage of shrub for each county (10%) | Continuous |
| Percentage coverage of grassland | Percentage coverage of grassland for each county (10%) | Continuous |
| Percentage coverage of dry field | Percentage coverage of dry field for each county (10%) | Continuous |
| Percentage coverage of paddy field | Percentage coverage of paddy field for each county (10%) | Continuous |
| Precipitation | Monthly mean of cumulative precipitation during the study period for each county (10mm) | Continuous |
| Temperature | Monthly average temperature during the study period for each county (℃) | Continuous |
| Relative humidity | Average relative humidity during the study period for each county (10%) | Continuous |
| Sunshine hours | Monthly average sunshine hours during the study period for each county (100 hours) | Continuous |
